# Persistence of KIR^neg^ NK cells after haploidentical hematopoietic stem cell transplantation protects from human cytomegalovirus infection/reactivation

**DOI:** 10.3389/fimmu.2023.1266051

**Published:** 2024-01-10

**Authors:** Clara Di Vito, Nicolò Coianiz, Michela Calvi, Sara Terzoli, Elisa Zaghi, Simone Puccio, Alessandro Frigo, Jacopo Mariotti, Chiara De Philippis, Daniele Mannina, Barbara Sarina, Rossana Mineri, Vu Thuy Khanh Le-Trilling, Mirko Trilling, Luca Castagna, Stefania Bramanti, Armando Santoro, Domenico Mavilio

**Affiliations:** ^1^ Unit of Clinical and Experimental Immunology, IRCCS Humanitas Research Hospital, Rozzano, Milan, Italy; ^2^ Department of Medical Biotechnologies and Translational Medicine (BioMeTra), University of Milan, Milan, Italy; ^3^ Department of Biomedical Sciences, Humanitas University, Milan, Italy; ^4^ Laboratory of Translational Immunology, IRCCS Humanitas Research Hospital, Milan, Italy; ^5^ Bone Marrow Transplant Unit, IRCCS Humanitas Research Hospital, Milan, Italy; ^6^ Molecular Biology Section, Clinical Investigation Laboratory, IRCCS Humanitas Research Hospital, Milan, Italy; ^7^ Institute for Virology, University Hospital Essen, University of Duisburg-Essen, Essen, Germany

**Keywords:** haploidentical hematopoietic stem cell transplantation, natural killer cells, immune reconstitution, human cytomegalovirus, viral immunity

## Abstract

Haploidentical hematopoietic stem cell transplantation (h-HSCT) is a therapeutic option to cure patients affected by hematologic malignancies. The kinetics and the quality of immune-reconstitution (IR) impact the clinical outcome of h-HSCT and limit the onset of life-threatening Human Cytomegalovirus (HCMV) infection/reactivation. Natural Killer (NK) cells are the first lymphocytes that recover after h-HSCT and they can provide rapid innate immune responses against opportunistic pathogens. By performing a longitudinal single-cell analysis of multiparametric flow-cytometry data, we show here that the persistence at high frequencies of CD158b1b2j^neg^/NKG2A^pos^/NKG2C^neg^/NKp30^pos^/NKp46^pos^ (KIR^neg^) NK cells is associated with HCMV infection/reactivation control. These KIR^neg^ NK cells are “unlicensed”, and are not terminal-differentiated lymphocytes appearing early during IR and mainly belonging to CD56^bright^/CD16^neg^ and CD56^bright^/CD16^pos^ subsets. KIR^neg^ NK cells are enriched in oxidative and glucose metabolism pathways, produce interferon-γ, and are endowed with potent antiviral activity against HCMV *ex vivo*. Decreased frequencies of KIR^neg^ NK cells early during IR are associated with clinically relevant HCMV replication. Taken together, our findings indicate that the prolonged persistence of KIR^neg^ NK cells after h-HSCT could serve as a biomarker to better predict HCMV infection/reactivation. This phenomenon also paves the way to optimize anti-viral immune responses by enriching post-transplant donor lymphocyte infusions with KIR^neg^ NK cells.

## 1 Introduction

Human cytomegalovirus (HCMV) is a β-herpesvirus that establishes lifelong latent infections and is estimated to affect about 60% of the western population. While the reactivation of HCMV is physiologically controlled by the immune system, it represents a severe condition in immune-compromised patients, including those receiving an allogeneic hematopoietic stem cell transplantation (HSCT) (1–5). Haploidentical HSCT (h-HSCT) with post-transplant Cyclophosphamide (PT-Cy) is one of the most effective platforms to cure hematologic malignancies and allows to find a donor for nearly every patient in need, including those aged 60 years or older, thanks to the reduced toxicity of the conditioning regimens (6, 7). In this context, the kinetics and quality of innate immune reconstitution (IR) emerged as predictors of transplant outcomes and adverse reaction onset (8). Natural Killer (NK) cells are the first lymphocytes to recover in the first month after h-HSCT and play essential roles in clearing residual leukemic cells and in limiting post-transplant complications (9–12). Despite the availability of diagnostics and antiviral drugs, productive HCMV replication, as a consequence of infection/reactivation (HCMV-I/R), represents one of the main life-threatening adverse events after h-HSCT (1).

HCMV greatly impacts NK cell homeostasis, maturation, and IR following HSCT by promoting the expansion of NKG2C^pos^ NK cells with adaptive properties (13–15). Accordingly, we previously confirmed that HCMV-I/R after h-HSCT also accelerates the maturation of NK cells in a h-HSCT setting. Moreover, we demonstrated that HCMV-I/R after h-HSCT is associated with the appearance of mature and adaptive-like CD158b1b2j^pos^/NKG2A^neg^/NKG2C^pos^/NKp30^lo^ NK cells that have impaired anti-viral effector-functions. The expansion of these adaptive-like and functionally impaired NK cells was associated not only with an increased reactivation of HCMV, but also with a higher probability of acute Graft versus Host Disease (GvHD) onset (16).

However, very little is known about the possible appearance or expansion of other NK cell subsets able to control viral infections soon after h-HSCT. Herein, by undertaking a longitudinal profile of immune-reconstituting NK cells by high-dimensional flow cytometry, we identified an NK cell subpopulation expanded early after h-HSCT that could protect h-HSCT recipients from HCMV-I/R.

## 2 Materials and methods

### 2.1 Patient recruitment and sample collection

This study included a total of 30 consecutive patients (**Table 1**) affected by hematologic malignancies who underwent a T-cell replete h-HSCT with post-transplant Cyclophosphamide (16) and their relative donors who were enrolled at the Bone Marrow Transplant Unit, IRCCS Humanitas Research Hospital, Rozzano, Milan, Italy between 2011 and 2016.

**Table 1:** Characteristics of patients enrolled in the study.

N	Sex: d/r	r: age	d: age	Disease; status before transplant	Conditioning regimen	HCMV d/r	HCMV-I/R (Y/N; day post-h-HSCT)	Follow-up (weeks; Reason for stopping)
1	F/F	20	51	HL; CR	Baltimore	-/-	N	53; End of the study
2	M/F	22	52	HL; CR	Baltimore	-/+	Y; 58	55; End of the study
3	F/M	53	47	NHL; PR	Baltimore	+/+	Y; 44	9; Deceased
4	F/F	33	61	HL; CR	Baltimore	+/+	N	55; End of the study
5	M/F	34	29	HL; CR	Baltimore	-/-	N	50; End of the study
6	M/M	25	51	NHL; CR	Baltimore	+/+	N	53; End of the study
7	F/M	47	38	HL; CR	Baltimore	+/+	Y; 33	47; End of the study
8	M/M	62	35	NHL; PR	Baltimore	+/-	Y; 62	50; End of the study
9	F/F	42	49	NHL; PR	Baltimore	+/+	Y; 65	54; End of the study
10	M/F	40	48	HL; PR	Baltimore	+/+	Y; 34	43; Deceased
11	M/F	21	52	HL; PR	Baltimore	-/-	N	9; Deceased
12	M/M	37	47	HL; PD	Baltimore	-/-	N	15; Deceased
13	F/M	33	64	AML; CR	TBF MAC	+/+	Y; 41	52; End of the study
14	F/M	35	27	ALL; CR	TBF MAC	+/+	Y; 39	21; Deceased
15	M/F	46	35	ALL; CR	TBF MAC	-/+	Y; 102	30; Other*
16	M/F	47	45	NHL; PD	Baltimore	+/+	Y; 50	11; Deceased
17	M/M	49	56	MDS; SD	TBF MAC	-/-	N	53; End of the study
18	M/M	38	53	HL; CR	ONC005	+/-	Y; 42	47; End of the study
19	F/F	28	29	HL; CR	GITMO	+/+	Y; 21	12; Consent withdrawal
20	M/M	70	32	MDS; CR	TBF RIC	+/+	Y; 39	6; Consent withdrawal
21	M/M	20	47	HL; PR	Baltimore	-/+	Y; 39	50; End of the study
22	M/M	25	28	HL; CR	Baltimore	+/-	Y; 40	40; Other*
23	M/M	70	37	MDS; SD	TBF RIC	-/+	Y; 31	52; End of the study
24	F/M	68	30	AML; CR	TBF RIC	+/+	Y; 28	20; Other*
25	F/M	36	40	ALL; CR	TBF MAC	+/-	Y; 48	47; End of the study
26	M/F	56	33	HL; CR	Baltimore	-/+	Y; 27	32; Other*
27	M/F	26	62	HL; CR	Baltimore	+/-	N	51; End of the study
28	F/F	48	41	MDS; CR	TBF RIC	-/-	N	35; Consent withdrawal
29	M/M	52	25	MF; PD	Baltimore	-/+	Y; 116	35; Other*
30	F/F	36	42	HL; PR	ONC005	-/-	N	43; Other*

d, donor; r, recipient; F, Female; M, Male; HL, Hodgkin Lymphoma; NHL, Non-Hodgkin Lymphoma; AML, Acute Myeloid Leukemia; ALL, Acute Lymphoid Leukemia; MDS, Myelodysplastic Syndrome; MF, Myelofibrosis: CR, complete response; PR, partial response; SD, stable disease; PD, progressive disease; Baltimore: Fludarabine (30mg/m2; days -6, -5, -4, -3, -2), Cyclophosphamide (14,5 mg/Kg; days -6, -5), Total Body Irradiation (200 cGy); RIC: Reduce Intensity Conditioning; MAC: Myeloablative Conditioning; TBF MAC: Thiotepa (5 mg/Kg; days -6, -5), Fludarabine (50 mg/m2; days -4, -3, -2), Busulphan (3.2 mg/Kg; days -4, -3, -2); TBF RIC: Thiotepa (5 mg/Kg; days -5), Fludarabine (50 mg/m2; days -4, -3, -2), Busulphan (3.2 mg/Kg; days -3, -2); ONC005: Thiotepa (5 mg/Kg twice a day; day -6), Fludarabine (30mg/m2; days -5; -4, -3, -2), Cyclophosphamide (30 mg/Kg; days -5), Total Body Irradiation (200 cGy); GITMO: Thiotepa (6 mg/Kg twice a day; day -5), Fludarabine (30mg/m2; days -4, -3), Cyclophosphamide (30 mg/Kg; days -4, -3); HCMV, Human Cytomegalovirus; HCMV-I/R, HCMV infection/reactivation; Y, yes; N, no; h-HSCT, haploidentical hematopoietic stem cell transplantation.

* patient stopped coming to the clinic.

Written informed consent was obtained from all patients and donors prior to sample collection, in accordance with the Declaration of Helsinki. Protocol approval was obtained from the Institutional Review Boards of the Clinical and Research Institute Humanitas (Approval 24/18).

The HCMV-I/R was evaluated in the peripheral blood of both donors and recipients before the transplant and monitored weekly in the recipients after h-HSCT by assessing the HCMV viral load through real-time PCR (CMV R-GENE, Argene, Biomérieux). When a viral load greater than 2,000 IU/mL was detected, recipients were considered as HCMV-reactivated. Preemptive therapy (Foscarnet 90 mg/kg or Ganciclovir 5 mg/kg and/or Valganciclovir, twice a day for 2 weeks) was given to reactivated patients with a viral load exceeding 4,000 IU/mL. All patients included in this study were enrolled before the introduction of Letermovir as HCMV prophylaxis.

Peripheral blood samples were obtained from both donors and recipients before h-HSCT, and from the recipients monthly until 1-year post-h-HSCT. Peripheral blood mononuclear cells (PBMCs) were isolated using standardized density gradient techniques (Lympholyte-H, Cedarlane) and frozen in liquid nitrogen according to standard procedures.

### 2.2 Polychromatic flow cytometry and PhenoGraph analysis

To minimize variability, the flow cytometry experiments were conducted in batches on frozen cells. Frozen cells were thawed and stained for 15 minutes at room temperature (RT) with Zombie Aqua™ Fixable Viability Kit (Biolegend) and 20 minutes at RT with fluorescent-conjugated monoclonal antibody mix (16).

Samples were acquired at BD FACSymphony A5 flow cytometer (BD Bioscience).

Flow Cytometry Standard (FCS) 3.0 files were imported into FlowJo software (version 9.9.6., TreeStar) and analyzed by standard manual gating strategy. NK cells were identified among viable single cells as Lineage^neg^ (CD3^neg^/CD14^neg^/CD4^neg^/CD15^neg^/CD20^neg^/CD19^neg^/CD33^neg^/CD34^neg^/CD203c^neg^/FCϵR^neg^) lymphocytes. Two thousand NK cells/sample were imported into FlowJo (v 10.2.0) to perform a biexponential transformation. Data were then exported in Python (v 3.7.3) and subjected to the Cytophenograph pipeline (http://github.com/luglilab/Cytophenograph) (16). The K-value was set at 45. New CSV files were then saved, and data were further analyzed in FlowJo (v 10.2.0). Clusters with a frequency lower than 0.5% were excluded from the analysis. Metaclustering was then performed using the gplots R package and PhenoGraph clusters were visualized using the Uniform Manifold Approximation and Projection (UMAP) dimension reduction technique.

### 2.3 *In vitro* HCMV infections

The EndoGRO HUVEC (MilliporeSigma) were cultured in cell flasks precoated with Collagen I (Corning) at 10^5^-10^6^ cells/mL by using the endothelial cell growth medium-2 (EGM-2) (Lonza) containing 5% FBS (Lonza), 1% Penicillin-Streptomycin (Invitrogen), and 1% UltraGlutamine (Lonza). The bacterial artificial chromosome (BAC) clone of the HCMV strain TB40/E expressing enhanced green fluorescent protein (EGFP) (17) was used to infect HUVEC at a multiplicity of infection (MOI) of 1 for 12 hours.

CD158b1b2j^neg^ NK cells were purified from PBMCs of NR and R recipients at 8-12 months after h-HSCT (**Supplementary Table 2**) by using a BD FACSAria III cell sorter (BD Bioscience). FACS-sorted CD158b1b2j^neg^ NK cells were then incubated overnight at 37°C in 5% CO_2_ in RPMI medium containing 10% FBS, 1% Penicillin-Streptomycin, and 1% UltraGlutamine (RPMI complete) and supplemented with 100 IU/mL rhIL-2 and 100 IU/mL rhIL-12. CD158b1b2j^neg^ NK cells were then cocultured with infected HUVECs for 72 hours in RPMI complete medium containing rhIL-2 (200 IU/mL). The images were acquired with the DMi8 Wide-Field Microscope (Leica). For each well, four representative fields were analyzed by counting the number of fluorescent-infected HUVECs by using ImageJ software (NIH).

### 2.4 Enzyme-linked immunosorbent assay

Interferon-γ (IFN-γ) production was quantified on the supernatant of purified CD158b1b2j^neg^ NK cells co-cultured for 72 hours in the presence of HCMV-infected HUVEC using the Human IFN-gamma DuoSet ELISA (R&D Systems), according to manufacturer’s instructions.

### 2.5 Total RNA sequencing

FACS-sorted CD158b1b2j^neg^ NK cells were lysed in 49 μL of RLT buffer (Qiagen) containing 1 μL of RNAse inhibitor (Thermo Fisher Scientific) and stored at -80°C.

The MicroRNAeasy KitTM with DNAse (Qiagen) was used to purify total RNA, which was then quantified by a Nanodrop 2000 (Thermo Fisher Scientific). Starting from 0.5 ng of high-quality total RNA with an RNA Integrity Number (RIN) greater than 6, assessed using a 4200 Tape Station (Agilent), libraries were prepared with the SMART-Seq Stranded Kit (Clontech-Takara). Libraries were then multiplexed in equimolar pools and sequenced by using a NextSeq-550 Illumina Platform. At least 60 million 75bp-paired-end reads per sample were generated. Read alignment, differential gene expression, and functional enrichment analyses were performed as previously described (16).

### 2.6 Statistics

GraphPad PRISM (version 9.0) and R statistical software were used to perform statistical analysis. Different variables among samples were compared by unpaired, two-tailed Student’s t-test. Different variables within the same sample were compared by using a paired, two-tailed Student’s t-test. Results are presented as the mean ± standard deviation (SD) or as the mean ± standard error of mean (SEM) when repeated sampling was considered. Two-sided or one-sided P values were considered significant when P ≤ 0.05.

## 3 Results

### 3.1 Patient characteristics

A total of 30 patients affected by hematologic diseases and receiving a T-cell replete h-HSCT with PT-Cy were enrolled in this study (**Table 1**; **Supplementary Table 1**). Peripheral blood mononuclear cells (PBMCs) were collected from recipients at different time points after transplant till one year after and from their relative HSC donors (16). HCMV-I/R was observed in 10/30 (33.3%) haplo-HSCT recipients with median onset at +40.5 days after h-HSCT (range 21-116).

Acute GvHD requiring systemic therapy was observed in 6/10 HCMV not-reactivated and 12/20 HCMV reactivated patients with a median day of onset at day 41 (range 19-104). Chronic GvHD was observed in 4/30 patients, three of whom belong to the HCMV reactivated group.

### 3.2 Longitudinal analysis of NK cells identifies NK cell clusters persisting in NR patients

**Figure 1:**
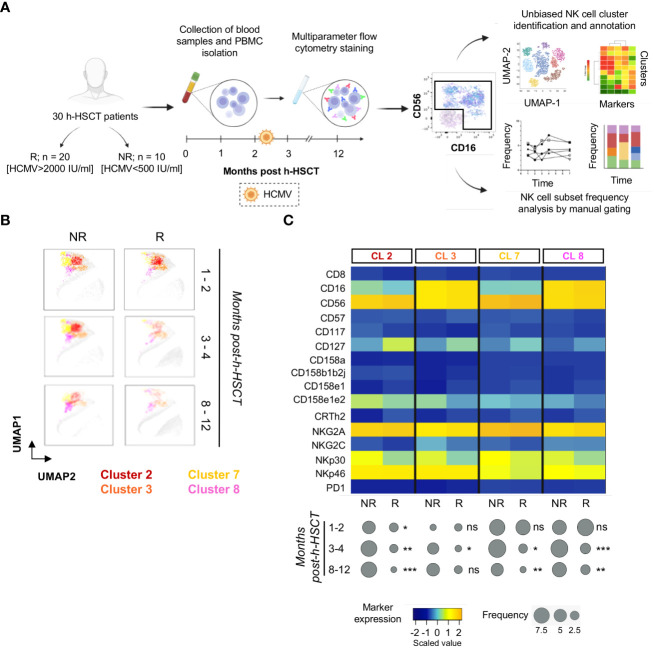
NK cells from HCMV not-reactivated recipients are characterized by a CD158b1b2j^neg^/NKG2A^pos^/NKG2C^neg^/NKp30^pos^/NKp46^pos^ phenotype. **(A)** Experimental workflow: peripheral blood mononuclear cells (PBMCs) were isolated from a total of 30 patients affected by hematologic diseases and receiving a T-cell replete h-HSCT with post-transplant Cyclophosphamide longitudinally for 1 year after h-HSCT. Patients were stratified by HCMV not-reactivated (NR) or reactivated (R). PBMCs were next stained and analyzed by multiparametric flow cytometry. NK cells, gated as viable Lineage^neg^ lymphocytes, were examined by PhenoGraph (upper panel), an unbiased method that uses marker expression to define the k-nearest neighbor for each single cell and then group and stratify cells in clusters, as well as by classical manual gating strategy (lower panel) to evaluate their phenotype and frequencies over time of the different NK cell subpopulations. **(B)** UMAP plots showing the distribution of clusters 2, 3, 7, and 8 in NR (left panels) and R (right panels) recipients before (1-2 months), soon after (3-4 months), and late after (8-12 months) HCMV infection/reactivation. NK cell clusters were overlaid with the total NK cell distribution (gray background). **(C)** Heatmap displaying the expression of NK cell markers on clusters (CL) 2, 3, 7, and 8 in NR (n=10) and in R (n=20) h-HSCT patients (upper panel). The median frequencies of each PhenoGraph NK cell cluster in NR and R patients at 1-2, 3-4, and 8-12 months after h-HSCT is shown in balloon plots (lower panel). Student's t-test NR vs R, *p<0.05, **p<0.01, ***p<0.001, ns: not significant.

We first performed a longitudinal analysis of high-dimensional flow cytometry data on circulating NK cells. To investigate the differences in NK cell immune reconstitution according to HCMV-I/R, h-HSCT recipients were stratified into two groups, namely either experiencing (R, n=20) or not (NR, n=10) the HCMV-I/R that generally occurs within the first 2-3 months after h-HSCT. NK cell data were next concatenated, analyzed with Cytophenograph pipeline, and visualized using Uniform Manifold Approximation and Projection (UMAP) (**Figure 1A**). These analyses identified 28 clusters of phenotypically distinct NK cells (16). We next focused our attention on four clusters (2, 3, 7, and 8) detectable at high frequencies during the first weeks after h-HSCT prior to HCMV-I/R in NR and R (**Figure 1B**). We observed that the percentages of these NK cell clusters started to decline in R at 3-4 months after h-HSCT, while their frequencies remained high in NR even after 12 months from the transplant (**Figure 1C**). A common phenotype of clusters 2, 3, 7, and 8 was the absence of Killer Immunoglobulin-like Receptors (KIR) CD158b1b2j, low expression of NKG2C, and high levels of NKG2A, NKp30, and NKp46.

### 3.3 CD158b1b2j^neg^/NKG2A^pos^/NKG2C^neg^/NKp30^pos^/NKp46^pos^ NK cells are expanded in NR patients

**Figure 2:**
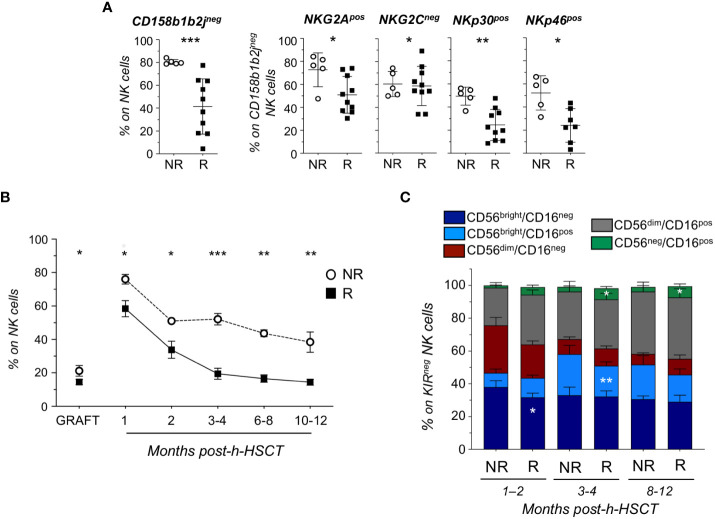
Longitudinal analysis reveals that KIR^neg^ NK cells persist in HCMV not-reactivated recipients up to 1-year post-h-HSCT and are mainly composed by less differentiated subsets. **(A)** Relative frequencies of CD158b1b2j^neg^ on total NK cells (left panel) and of NKG2A^pos^, NKG2C^neg^, NKp30^pos^, and NKp46^pos^ on CD158b1b2j^neg^ NK cells in NR (○; n=5) and R (▪; n=10) recipients at 8-12 months after h-HSCT. **(B)** Summary statistical graph depicting the frequency (%, mean ± SEM) of CD158b1b2j^neg^/NKG2A^pos^/NKG2C^neg^/NKp30^pos^/NKp46^pos^ NK cells on total NK cells in NR (○; n=10) and R (▪; n=20) recipients and their grafts (NR=6; R=13) at 1, 2, 3-4, 6-8, and 10-12 months after the h-HSCT. **(C)** Histograms showing the NK cell subset distribution (%; mean ± SD) within CD158b1b2j^neg^/NKG2A^pos^/NKG2C^neg^/NKp30^pos^/NKp46^pos^ (KIR^neg^) NK cells in NR and R at 1-2, 3-4, and 8-12 months after h-HSCT. Student’s t-test NR vs R, *p<0.05, **p<0.01, ***p<0.001.

The manual gating strategy analysis confirmed that CD158b1b2j^neg^ NK cells were significantly enriched in NR patients, who also expressed high levels of NKG2A, NKp30, and NKp46 and lower levels of NKG2C (**Figure 2A**).

By combining these markers differentially expressed in R and NR, we identified a novel CD158b1b2j^neg^/NKG2A^pos^/NKG2C^neg^/NKp30^pos^/NKp46^pos^ (KIR^neg^) NK cell subset. This NK cell subpopulation was detectable at significantly higher frequencies in NR compared to R at all time points analyzed. Moreover, the percentage of KIR^neg^ NK cells started to decrease in R after 2 months from the transplant and further declined after the onset of HCMV-I/R. Conversely, circulating KIR^neg^ NK cells persisted more in NR compared to R even 12 months after the transplant (**Figure 2B**; **Supplementary Figure 1**). Of note, we also found significantly higher amounts of KIR^neg^ NK cells in the grafts of NR compared to those of R (**Figure 2B**), regardless of their HCMV serostatus and donor age (**Supplementary Figure 2**).

By assessing the NK cell subset distribution among CD158b1b2j^neg^/NKG2A^pos^/NKG2C^neg^/NKp30^pos^/NKp46^pos^ NK cells, we observed that they mainly belonged to less mature CD56^bright^/CD16^neg^, CD56^bright^/CD16^pos^, and CD56^dim^/CD16^neg^ NK cell subsets in both NR and R and at all time points considered (10, 18). Moreover, we demonstrated that CD56^bright^/CD16^neg^ and CD56^bright^/CD16^pos^ NK cells with a CD158b1b2j^neg^/NKG2A^pos^/NKG2C^neg^/NKp30^pos^/NKp46^pos^ phenotype were present at significantly higher frequencies in NR compared to R at 1-2 months and 3-4 months after h-HSCT, respectively. In clear contrast and in line with previous findings (8, 19), our data showed that HCMV-I/R induced a significant expansion of terminally differentiated CD56^neg^/CD16^pos^ NK cells in R, considering both total and KIR^neg^ NK cells (**Figure 2C**; **Supplementary Figure 3A**).

**Figure 3:**
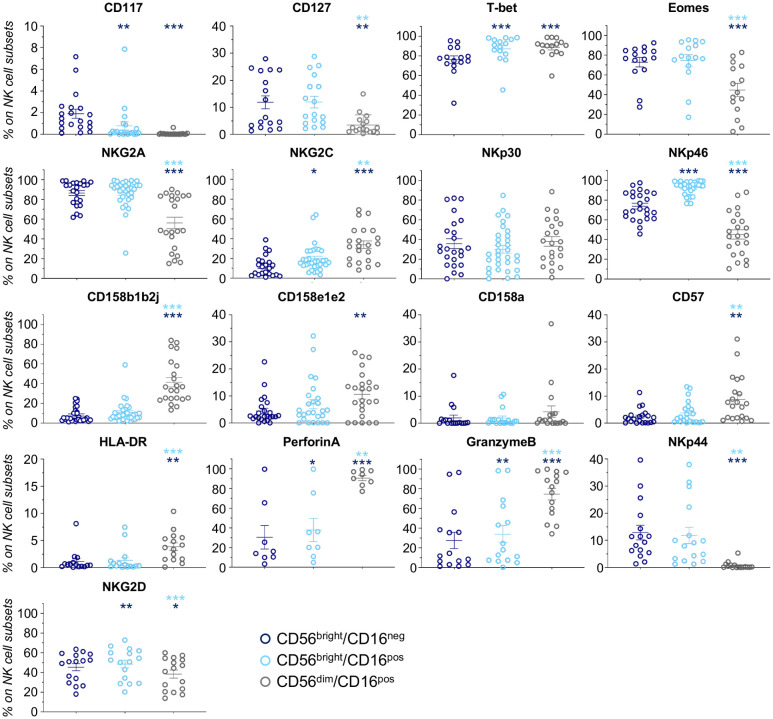
NK cell markers in CD56^bright^/CD16^neg^, CD56^bright^/CD16^pos^, and CD56^dim^/CD16^pos^ NK cells. Scatter plots showing expression (%; mean ± SEM) of markers involved in NK cell differentiation (CD117, CD127, T-bet, and Eomes), maturation/licensing (NKG2A, NKG2C, CD158b1b2j, CD158e1e2, CD158a, and CD57), activation (NKp44 and HLA-DR), and function (PerforinA, GranzymeB, NKp30, and NKp46) in h-HSCT patients at 3-4 months after the transplant. CD56^bright^/CD16^neg^ (blue) CD56^bright^/CD16^pos^ (light blue) and CD56^dim^/CD16^pos^ (gray) Paired t-test, *p<0.05, **p<0.01, ***p<0.001 (light blue vs CD56^bright^/CD16^neg^, blue vs CD56^bright^/CD16^pos^).

We next focused our attention on CD56^bright^/CD16^pos^ NK cells, a neglected subset of unknown origin, which is present at very low frequency under homeostatic conditions or during post-h-HSCT IR (10, 18, 20). Our data demonstrated that CD56^bright^/CD16^pos^ NK cell frequencies and absolute counts were significantly higher in NR in the first months after the transplant (**Figure 2C**; **Supplementary Figures 3A, B**). Phenotypic features of CD56^bright^/CD16^pos^ NK cells place them in an intermediary maturation stage between CD56^bright^/CD16^neg^ and CD56^dim^/CD16^pos^ in term of differentiation markers (CD117, CD127, T-bet, EOMES, and CD57), KIRs (CD158b1b2j and CD158e1e2), activating and inhibitory receptors (NKG2A, NKG2C, NKp30, NKp46, and NKp44), and cytolytic potential (PerforinA and GranzymeB) (**Figure 3**).

### 3.4 KIR^neg^ NK cells show a higher anti-viral potential

**Figure 4:**
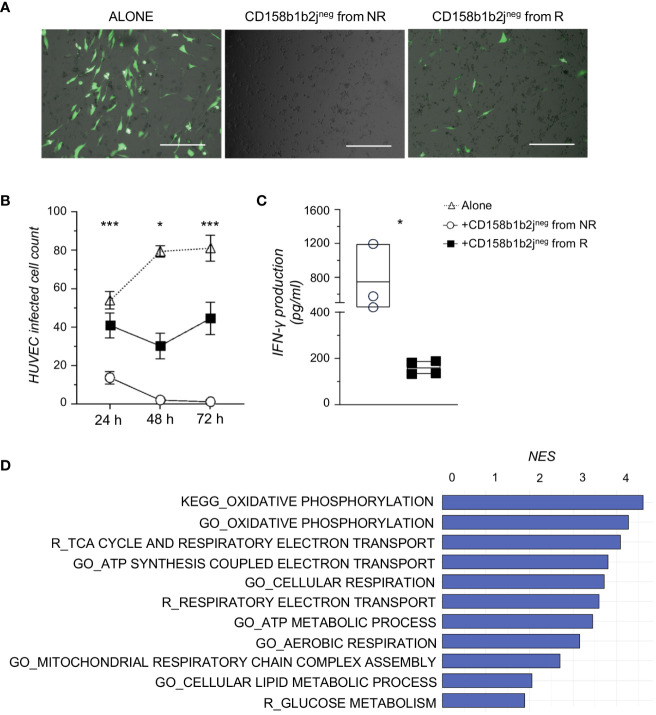
CD158b1b2j^neg^ NK cells from NR better control HCMV infection and have higher metabolic functions than those from R. **(A)** Representative overlayed images of immunofluorescence and bright field pictures of HUVEC infected with TB40:EGFP (MOI=1), a BAC clone of the HCMV strain TB40/E expressing EGFP, and co-cultured for 48 hours alone (left panel) or in the presence of CD158b1b2j^neg^ NK cells from NR (middle panel) or R (right panel). Scale bar 400 μm. **(B)** Summary statistical graph displaying the number of TB40:EGFP-infected HUVEC remained after 24, 48, and 72 hours **(h)** of culture alone or in the presence of CD158b1b2j^neg^ NK cells from NR (n=4) or R (n=4) patients. Student’s t-test NR vs R, *p<0.05, **p<0.01, ***p<0.001. **(C)** Summary statistical graph showing the IFN-γ production (pg/ml) by purified CD158b1b2j^neg^ NK cells from NR (o) and R (▪) after 72 hours of co-culture with TB40:Egfp HUVEC-infected cells and assessed using the Human IFN-gamma DuoSet ELISA (R&D Systems). Student’s t-testNR vs R, *p<0.05. **(D)** Gene sets, with a False Discovery Rate < 0.05 and involved in energy metabolism, more enriched in CD158b1b2j^neg^ NK cells from NR compared to R as retrieved from Gene Ontology (GO_), KEGG, and Reactome (R_) databases. NES=Normalized Enrichment Score.

To assess the functional relevance of KIR^neg^ NK cells in controlling HCMV infection, we investigated their ability to prevent HCMV replication *ex vivo* (16, 20). To obtain enough purified NK cells that should recapitulate those NK cell clusters highly enriched in NR, CD158b1b2j^neg^ NK cells were FACS-sorted from NR (n=4) and R (n=4) at 8-12 months (**Supplementary Table 2**) and co-cultured with HUVEC cells infected with the BAC clone of the HCMV strain TB40/E expressing EGFP for a maximum of 72 hours. Every 24 hours cells were observed with a microscope and the number of green infected HUVEC cells was counted. Our results first showed that CD158b1b2j^neg^ NK cells diminished the HCMV spread in permissive endothelial cells. Of note, CD158b1b2j^neg^ NK cells from NR were able to completely suppress HCMV-infection *ex vivo* (**Figures 4A, B**). Accordingly, our data also demonstrated that CD158b1b2j^neg^ NK cells purified from NR were able to release higher amounts of INF-γ after co-culture with HCMV-infected HUVEC compared to R (**Figure 4C**).

To better assess the functional properties of our newly disclosed NK cell subset, we investigated the transcriptional profile of purified CD158b1b2j^neg^ NK cells from NR and R by RNA sequencing. Our analyses identified 1327 differentially expressed genes (DEGs; p-value <0.05), 72 of which had a p-value adjusted<0.05. We next performed a Gene Set Enrichment Analysis (GSEA) and found that CD158b1b2j^neg^ NK cells from NR were more enriched in pathways associated with oxidative and glucose metabolisms compared to R (**Figure 4D**).

## 4 Discussion

Haploidentical HSCT with PT-Cy represents a therapeutic platform with curative potential for patients affected by high-risk hematologic malignancies and enables the rapid sourcing of a donor for almost every patient in need. Due to the prolonged immunodeficiency post-h-HSCT, the clinical outcome is still hampered by opportunistic viral infections, mainly caused by new infections or from reactivations of HCMV (21).

In a h-HSCT setting, we and others previously reported that NK cells are the first donor-derived lymphocytes to immune-reconstitute, thus conferring a certain degree of immune protection against pathogens and leukemic cells early after transplant (10–12).

Moreover, we and others demonstrated that, in patients who underwent h-HSCT, the homeostasis of NK cells is influenced by the occurrence of HCMV-I/R, which accelerates the maturation of NK cells and drives the expansion of NKG2C^pos^ NK cells endowed with adaptive traits (15). In this context, a recent study investigating the NKG2C genotype as a factor predicting the h-HSCT outcome showed that NKG2C homozygosity in the donor ameliorates HCMV clearance in the recipient and promotes the quantitative and qualitative reconstitution of adaptive NKG2C^pos^ NK cells (22). On the other hand, we demonstrated that HCMV-I/R can also mediate the expansion of dysfunctional CD158b1b2j^pos^/NKG2A^neg^/NKG2C^pos^/NKp30^lo^ NK cells (16).

Herein, we identified a novel CD158b1b2j^neg^/NKG2A^pos^/NKG2C^neg^/NKp30^pos^/NKp46^pos^ NK cell subset persisting in HCMV not-reactivated patients and characterized by a strong anti-viral potential.

Our unbiased computational analysis of single cell flow cytometry data demonstrated that the frequencies and absolute counts of KIR^neg^ NK cells are higher in NR than in R before HCMV-I/R occurrence. These KIR^neg^ NK cells further decline in R after the occurrence of HCMV-I/R because of the expansion of KIR^pos^ adaptive NK cells in R at the expense of KIR^neg^ ones (15, 16). Of note, we also found significantly higher amounts of KIR^neg^ NK cells in the grafts of NR compared to those of R, regardless of their HCMV serostatus and donor age. This observation could be of clinical utility since lower frequencies of KIR^neg^ NK cells in potential familiar donors may be predictive of HCMV-I/R in the recipient. Thus, the assessment of KIR^neg^ NK cells’ frequencies can be implemented, together with other NK cell properties, including the NKG2C genotype (22), KIR genotype, and alloreactivity (23, 24), for the screening of patient relatives to select optimal donors whose graft helps to prevent post-transplant HCMV-I/R and to ameliorate the clinical outcome of recipients in terms of engraftment, fast immune-reconstitution, and prevention of disease relapse and GvHD onset.

In contrast to mature CD158b1b2j^pos^/NKG2A^neg^/NKG2C^pos^/NKp30^lo^ NK cells expanded in R, which are mainly composed by CD56^dim^/CD16^pos^ and CD56^neg^/CD16^pos^ NK cell subsets (16), KIR^neg^ NK cells are in earlier stages of differentiation as they mainly belong to CD56^bright^/CD16^neg^ and CD56^bright^/CD16^pos^ subsets. Of particular significance are CD56^bright^/CD16^pos^ NK cells, a neglected subset of unknown origin, which is present at very low frequency under homeostatic conditions or during post-h-HSCT IR (10, 18, 20). By investigating the expression of markers involved in NK cell differentiation, maturation/licensing, activation, and function, we herein demonstrated that CD56^bright^/CD16^pos^ NK cells are an intermediate step of differentiation between CD56^bright^/CD16^neg^ and CD56^dim^/CD16^pos^ NK cells rather than CD56^dim^/CD16^pos^ NK cells upregulating CD56 in response to the cytokine storm occurring in patients undergoing h-HSCT (20, 25).

In line with previous findings made in a CMV mouse model (26), our data reveal in a clinical setting that “not terminally-differentiated” KIR^neg^ NK cells are involved in protection from HCMV-I/R. Indeed, the persistence of KIR^neg^ NK cells in NR till 1-year post-h-HSCT indicates that they could participate in efficient HCMV control following h-HSCT. On the contrary, KIR^neg^ NK cells decline in R after HCMV-I/R onset, suggesting that the decreased frequencies of these NK cells, together with the parallel increase of KIR^pos^ NK cells (16), could predispose an individual to HCMV-I/R and could predict the onset of this opportunistic condition.

In agreement, our *ex vivo* model of HCMV infection clearly demonstrated that, contrary to CD158b1b2j^pos^ NK cells (16), CD158b1b2j^neg^ NK cells from both NR and R limited the HCMV spread in permissive endothelial cells, even at 1-year after the transplant and far from the reactivation events. Of note, CD158b1b2j^neg^ NK cells from NR completely abolish HCMV-infection. This could be due to the higher NKG2A-education of CD158b1b2j^neg^ NK cells from R compared to NR, as well as to the higher expression of the natural cytotoxicity receptors NKp30 and NKp46 (27). In accordance, we also found that INF-γ production was significantly higher in CD158b1b2j^neg^ NK cells purified from NR compared to R, thus corroborating once more the anti-viral ability of NR-derived KIR^neg^ NK cells.

Recent findings indicate that cellular metabolism could determine the functional differences in NK cells and that an enhanced functional activity has been associated with the NK cell’s ability to efficiently utilize glucose to fuel glycolysis and oxidative phosphorylation (27). In agreement, our RNA-sequencing analyses clearly demonstrated that CD158b1b2j^neg^ NK cells from NR are characterized by a superior metabolic function that could be responsible for the enhanced anti-viral activity compared to CD158b1b2j^neg^ NK cells from R.

In summary, we show here that the chronic persistence of KIR^neg^ NK cells in h-HSCT patients is associated with HCMV control. This phenomenon is due to the higher anti-viral potential and superior metabolic functions of this NK cell subset. These “not terminally-differentiated” and “NKG2A educated” KIR^neg^ NK cells can persist for several months in NR, while keeping high surface levels of the checkpoint inhibitor NKG2A. Further investigations are warranted to identify the mechanism(s) ensuring the persistence and the homeostasis of KIR^neg^ NK cells in NR and to address if the NKG2A blockade may further improve their anti-viral potential (10). Moreover, although the data obtained could have a clinical utility in predicting the onset of HCMV-I/R and in potentially identifying the best donor among patient relatives whose graft helps to prevent post-transplant HCMV-I/R, to confirm and validate our findings a larger prospective study is necessary to allow patient stratification based on the disease stage and category, HCMV serostatus, and HCMV-I/R severity. Our findings may enable the optimization of post-transplant Donor-Lymphocyte Infusions by enriching or selecting “unlicensed” KIR^neg^ NK cells for superior HCMV-I/R control.

## Data availability statement

The datasets presented in this study can be found in online repositories. The names of the repository/repositories and accession number(s) can be found below: https://www.ncbi.nlm.nih.gov/geo/, GSE160362.

## Ethics statement

The studies involving humans were approved by Institutional Review Boards of the Clinical and Research Institute Humanitas (Approval 24/18) Comitato etico indipendente IRCCS Istituto Clinico Humanitas. The studies were conducted in accordance with the local legislation and institutional requirements. The participants provided their written informed consent to participate in this study.

## Author contributions

CDV: Conceptualization, Formal Analysis, Investigation, Methodology, Supervision, Writing – original draft, Writing – review & editing, Project administration. NC: Formal Analysis, Investigation, Methodology, Writing – original draft. MC: Formal Analysis, Investigation, Methodology, Writing – review & editing. ST: Data curation, Formal Analysis, Writing – review & editing. EZ: Formal Analysis, Investigation, Methodology, Writing – review & editing. SP: Data curation, Formal Analysis, Software, Writing – review & editing. AF: Investigation, Methodology, Writing – review & editing. JM: Resources, Writing – review & editing. CP: Resources, Writing – review & editing. DaM: Resources, Writing – review & editing. BS: Resources, Writing – review & editing. RM: Resources, Writing – review & editing. VTKL-T: Methodology, Resources, Writing – review & editing. MT: Funding acquisition, Methodology, Resources, Writing – review & editing. LC: Resources, Writing – review & editing. SB: Resources, Writing – review & editing. AS: Resources, Supervision, Writing – review & editing. DoM: Formal Analysis, Funding acquisition, Project administration, Supervision, Writing – original draft, Writing – review & editing.

## Supplementary material

Supplementary Table 1 Statistical analysis of NR and R patient characteristics. (related to Table 1).

Supplementary Table 2 Characteristics of patients used in *in vitro* infection experiments. (related to *in vitro* infection experiments).

Supplementary Figure 1 Longitudinal analysis of KIR^neg^ NK cell absolute counts in NR and R. (related to Figure 2B).

Supplementary Figure 2 KIR^neg^ NK cell frequency in donor grafts. (related to Figure 2B).

Supplementary Figure 3 NK cell subset distribution in recipients experiencing or not a HCMV-I/R after h-HSCT. (related to Figure 2C).
