# Supplementary material for: Persistence of KIRneg NK cells after haploidentical hematopoietic stem cell transplantation protects from human cytomegalovirus infection/reactivation
Source: Front Immunol. 2024 Jan 10;14:1266051. doi: 10.3389/fimmu.2023.1266051 (PMC10806243; doi:10.3389/fimmu.2023.1266051)
Supplement: Supplementary Table 1 — Statistical analysis of NR and R patient characteristics. (related to Table 1). [file DataSheet_1.pdf]

Research topic: The immune microenvironment and the infectious complications of allo-HSCT

Persistence of KIR^{neg} NK cells after haploidentical hematopoietic stem cell transplantation protects from human Cytomegalovirus infection/reactivation

Clara Di Vito[1*], Nicolò Coianiz[1], Michela Calvi[1,2], Sara Terzoli[1,3], Elisa Zaghi[1], Simone Puccio[4], Alessandro Frigo[1,2], Jacopo Mariotti[5], Chiara De Philippis[5], Daniele Mannina[5], Barbara Sarina[5], Rossana Mineri[6], Vu Thuy Khanh Le-Trilling[7], Mirko Trilling[7], Luca Castagna[5], Stefania Bramanti[5], Armando Santoro[5], Domenico Mavilio[1,2*].

[1]Unit of Clinical and Experimental Immunology, IRCCS Humanitas Research Hospital, Rozzano, Milan, Italy.

[2]Department of Medical Biotechnologies and Translational Medicine (BioMeTra), University of Milan, Milan, Italy.

[3]Department of Biomedical Sciences, Humanitas University, Rozzano, Milan, Italy.

[4]Laboratory of Translational Immunology, IRCCS Humanitas Research Hospital, Rozzano, Milan, Italy.

[5]Bone Marrow Transplant Unit, IRCCS Humanitas Research Hospital, Rozzano, Milan, Italy.

[6]Molecular Biology Section, Clinical Investigation Laboratory, IRCCS Humanitas Research Hospital, Milan, Italy.

[7]Institute for Virology, University Hospital Essen, University of Duisburg-Essen, Essen, Germany.

*** Correspondence:**

Clara Di Vito

clara.di_vito@humanitasresearch.it

Domenico Mavilio

domenico.mavilio@unimi.it; domenico.mavilio@humanitas.it

Supplementary Material

Supplementary tables

Supplementary table 1. Statistical analysis of NR and R patient characteristics.

	NR (n; %)	R (n; %)	P value (χ^2)
**Donor M**	6; 60%	12; 60%	1
**Donor F**	4; 40%	8; 40%	
**Recipient M**	7; 70%	13; 65%	0.78
**Recipient F**	3; 30%	7; 35%	
**Donor HCMV -**	7; 70%	6; 30%	0.37
**Donor HCMV +**	3; 30%	14; 70%	
**Recipient HCMV -**	8; 80%	4; 20%	0.001***
**Recipient HCMV +**	2; 20%	16; 80%	
**Acute GvHD YES**	6; 60%	12; 60%	0.69
**Acute GvHD NO**	4; 40%	8; 40%	
**Chronic GvHD YES**	1; 10%	3; 15%	
**Chronic GvHD NO**	9; 90%	17; 85%	0.70
	**NR (median; range)**	**R (median; range)**	**P value** (Mann-Whitney test)
**Donor age**	51; 29-62	37.5; 25-64	0.027*
**Recipient age**	33.5; 20-49	44; 20-70	0.053

Abbreviations: NR, not-reactivated patients; R, reactivated patients; F, Female; M, Male; GvHD, Graft versus Host Disease.

Supplementary table 2. Characteristics of patients used in *in vitro* infection experiments.

**Group**	**Day post-h-HSCT** used for in vitro infection experiments	**HCMV d/r**	**HCMV-I/R** (day post-h-HSCT)	**Sex**: d/r	**r: age**	**d: age**	**Disease; status before transplant**	**Follow-up** (weeks)
NR	369	+/+	NA	M/M	25	51	NHL; CR	53
NR	231	+/-	NA	M/F	26	62	HL; CR	51
NR	228	+/+	NA	F/M	54	55	NHL; SD	51
NR	377	-/-	NA	M/M	68	32	MDS; CR	54
R	331	+/+	33	F/M	47	38	HL; CR	47
R	258	+/-	62	M/M	62	35	NHL; PR	50
R	241	-/+	31	M/M	70	37	MDS; SD	52
R	362	+/+	54	M/M	52	50	HL; CR	52

Abbreviations: NR, not-reactivated patients; R, reactivated patients; d, donor; r, recipient; F, Female; M, Male; NHL, Non-Hodgkin Lymphoma; HL, Hodgkin Lymphoma; MDS, Myelodysplastic Syndrome; CR, complete response; SD, stable disease; PR, partial response.

Supplementary Figures

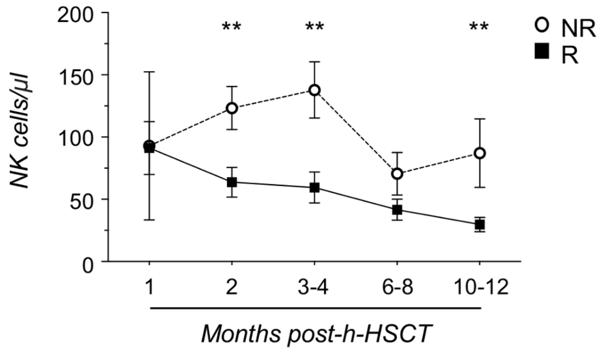

Supplementary Figure 1. Longitudinal analysis of KIR^{neg} NK cell absolute counts in NR and R.
Summary statistical graphs showing the absolute counts (NK cells/μl, mean ± SD) of CD158b1b2j^{neg}/NKG2A^{pos}/NKG2C^{neg}/NKp30^{pos}/NKp46^{pos} NK cells in NR (○; n=10) and R (■; n=20) recipients at 1, 2, 3-4, 6-8, 10-12 months after the h-HSCT. Student's t-test NR vs R, **p<0.01.

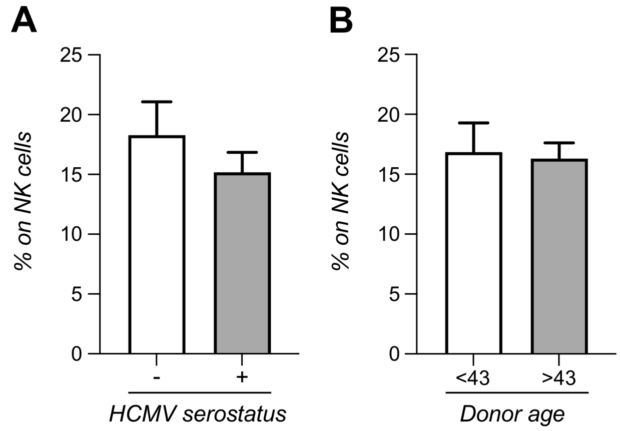

Supplementary Figure 2. KIR^{neg} NK cell frequency in donor grafts.
Summary statistical graphs showing the frequency (%, mean ± SD) of CD158b1b2j^{neg}/NKG2A^{pos}/NKG2C^{neg}/NKp30^{pos}/NKp46^{pos} NK cells on total NK cells in donor grafts subdivided based on **(A)** their HCMV serostatus or **(B)** age. The cut-off age of 43 corresponds to the median age of donors. Student's t-test, not significant.

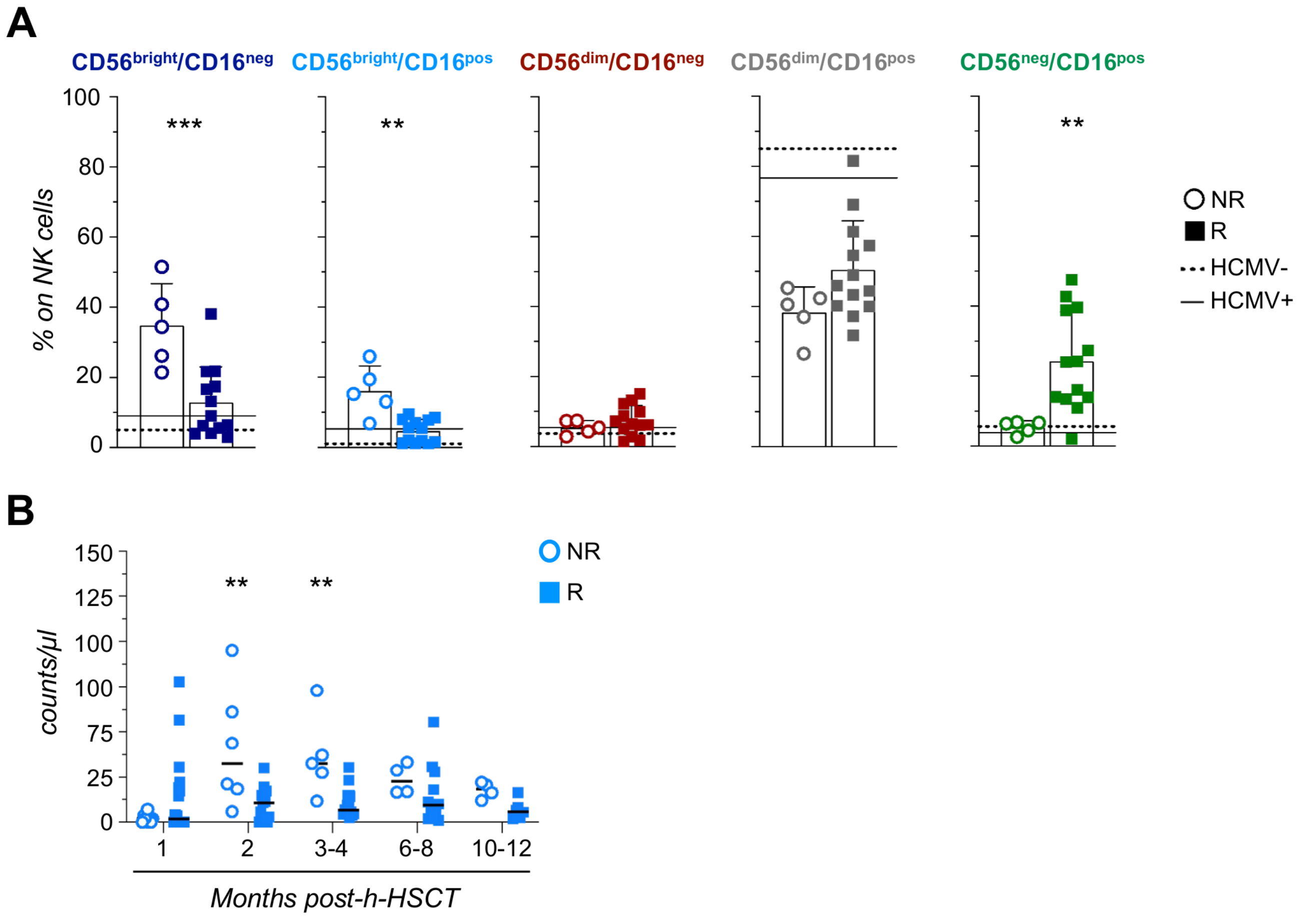

Supplementary Figure 3. NK cell subset distribution in recipients experiencing or not a HCMV infection/reactivation after h-HSCT.

(A) Summary statistical graphs showing the NK cell subset distribution (%; mean ± SD) in NR (○; n=5) and R (■; n=20) recipients on total NK cells at 3-4 months after h-HSCT. Solid and dotted lines represent the average frequency of NK cell subsets in HCMV seropositive (HCMV+, n=5) and seronegative (HCMV-, n=5) healthy donors, respectively.

(B) Summary statistical graphs showing the absolute counts (cells/µL, line at mean) of circulating CD56^{bright}/CD16^{pos} NK cells in recipients either experiencing (R: ■; n=20) or not (NR: ○; n=10) HCMV reactivation/infection at different time points after h-HSCT.

Student's t-test NR vs R, *p<0.05, **p<0.01, ***p<0.001.
